# Summer Course of Medical Lectures

**Published:** 1844-03

**Authors:** 


### SUMMER COURSE OF MEDICAL LECTURES.

Several members of the Faculty of the Medical Institute have associated themselves with a number of the physicians of Louisville, for the purpose of conducting a course of Lectures in the city, on all the departments of medicine, during the summer. The course will commence on the 1st of April, and be continued until the 1st of November, with a vacation during the months of July and August. An excellent opportunity will be afforded by the course to students for acquiring practical knowledge, of which it is expected that many will avail themselves. Y.

